# Complex Variant t(9;22) Chromosome Translocations in Five Cases of Chronic Myeloid Leukemia

**DOI:** 10.1155/2009/187125

**Published:** 2009-07-28

**Authors:** Ana Valencia, José Cervera, Esperanza Such, Eva Barragán, Pascual Bolufer, Oscar Fuster, Rosa Collado, Jesús Martínez, Miguel A. Sanz

**Affiliations:** ^1^Department of Hematology, La Fe University Hospital, 46009 Valencia, Spain; ^2^Molecular Biology Laboratory, Department of Medical Biopathology, La Fe University Hospital, 46009 Valencia, Spain; ^3^Department of Hematology, Hospital General Universitario de Valencia, 46014 Valencia, Spain

## Abstract

The Philadelphia (Ph^1^) chromosome arising from the reciprocal t(9;22) translocation is found in more than 90% of chronic myeloid leukemia (CML) patients and results in the formation of the chimeric fusion gene *BCR-ABL*. However, a small proportion of patients with CML have simple or complex variants of this translocation, involving various breakpoints in addition to 9q34 and 22q11. We report five CML cases carrying variant Ph translocations involving both chromosomes 9 and 22 as well as chromosomes 3, 5, 7, 8, or 10. G-banding showed a reciprocal three-way translocation involving 3q21, 5q31, 7q32, 8q24, and 10q22 bands. *BCR-ABL* fusion signal on der(22) was found in all of the cases by FISH.

## 1. Introduction

Chronic myelogenous leukemia (CML) is characterized by the Philadelphia chromosome (Ph^1^), resulting from a balanced translocation between the long arms of chromosome 9 and 22, the t(9;22)(q34;q11.2) [1]. In the formation of the Ph^1^ chromosome, the 3′ region of the *c-ABL* oncogene is transposed from 9q34 to the 5′ region of the *BCR* gene on chromosome 22 to form a fusion gene *BCR-ABL*, which encodes a fusion protein with constitutive tyrosine kinase activity [2]. Although the vast majority of patients with CML show the classical t(9;22)(q34;q11.2) translocation, variant Ph translocations are present in 5%–10% of CML cases. These are cytogenetically classified as simple variants involving chromosome 22 and a chromosome other than 9, and complex variants that involve chromosomes 9, 22, and one or more other chromosomes [3]. In almost all the cases with variant Ph^1^ chromosome, the *BCR-ABL* rearrangement can be detected by molecular methods or by fluorescence in situ hybridization (FISH). 

In this work, we described five patients diagnosed with CML carrying different complex variant Ph translocations involving chromosomes 9, 22 as well as one other chromosome. They were studied by G-banding, FISH, and reverse transcription-polymerase chain reaction (RT-PCR).

## 2. Material and Methods

### 2.1. Patients

Between March 1999 and November 2005, 81 CML patients were diagnosed in our laboratory. Informed consent was obtained from the patient or the patient's guardians in accordance with the Declaration of Helsinki, and the study was approved by the local ethical committee. Of them, five patients (6%) showed variant Ph translocations involving chromosomes 3, 5, 7, 8, or 10. Main clinical characteristics of these patients are presented in Table 1.

### 2.2. Cytogenetic Study

Conventional cytogenetic analysis was performed on unstimulated 24-hour culture of a bone marrow (BM) specimen. The cells were cultured and processed by conventional methods, and the chromosomes were stained with trypsin-Giemsa banding (GTG-banding). The karyotype was described according to the International System for Human Cytogenetic Nomenclature (ISCN, 2005) [4].

### 2.3. FISH Analysis

FISH analysis was performed on prepared slides of methanol/acetic-fixed BM cells using the *BCR/ABL* extra signal (ES) dual-color probe kit (Vysis Inc., Downers Grove, IL). Briefly, fresh slides were prepared from the cytogenetic pellet stored in fixative at −20°C and dehydrated with ethanol. Probes and slides were codenatured at 75°C for 1 minute and cohybridized overnight at 37°C in a HYBrite denaturation/hybridization system (Vysis). Slides were washed and counterstained with 4′-6-diamidino-2-phenylindole (DAPI) stain. Fluorescent signals were visualized under a Nikon Eclipse E600 microscope (Nikon, Tokyo, Japan) equipped with a CCD camera and analyzed using ISIS image analysis software (Metasystems Inc., Germany).

### 2.4. Quantitative, Real-Time, Reverse Transcriptase Polymerase Chain Reaction (QT-RT-PCR) of the Chimeric BCR-ABL Transcript

RNA was extracted from BM cells of the patient using the MagNa Pure LC mRNA HS kit (Roche Diagnostics GmbH Mannheim, Germany) automated on the MagNa Pure robot (Roche Diagnostics GmbH Mannheim). Reverse transcription was performed in a final volume of 25 *μ*L, following the manufacturer's instructions (TaqMan Reverse Transcription Reagents, Applied Biosystems, Foster City, CA). After cDNA synthesis, QT-RT-PCR was performed to detect chimeric transcripts derived from the translocation t(9;22). QT-RT-PCR assays were carried out with LightCycler (Roche Diagnostics GmbH Mannheim), using LightCycler Fast Start DNA Master hybridization Probes (Roche Diagnostics GmbH Mannheim). For detecting *BCR/ABL* fusion transcript, the samples were analyzed using the primers and specific labeled probes described by Bolufer et al. [5]. *BCR/ABL* amplified products were normalized to *ABL* amplifications for each sample using the primers A2 and CA3 described by Cross et al. [6].

## 3. Results

The group of five patients consisted of 3 females and 2 males, ranging in age at diagnosis from 50 to 75 years. All the patients were in chronic phase at presentation and were treated accordingly to what was considered the standard treatment in each moment receiving hydroxyurea, interferon-*α* and imatinib. One patient underwent autologous peripheral blood stem cell transplantation after failure of interferon-*α*. 

**Figure 1:**
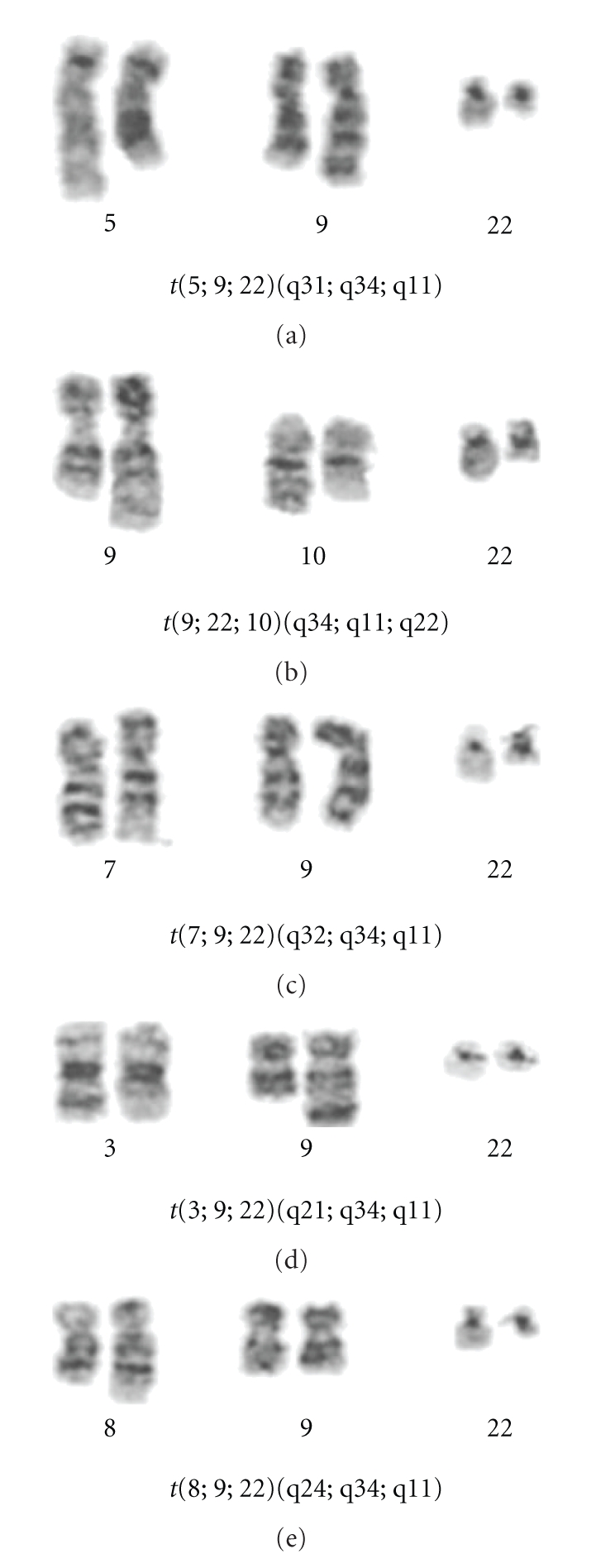
Partial karyotypes of the variant translocations. The letters correspond to the various patient cases.

Cytogenetic analysis by G-banding revealed the presence of five reciprocal three-way variant translocations of the classical t(9;22)(q34;q11). The chromosome breakpoints involved in these complex variant translocations were the following: 3q21, 5q31, 7q32, 10q22 and 8q24 (Figure 1). In addition, patients (a) and (d) also present additional abnormalities: patient (a) showed a complex karyotype with at least two main unrelated clones, whereas patient (d) showed numerical abnormalities of chromosomes 15 and 22. *BCR/ABL* dual-color FISH demonstrated in all cases the usual pattern of *BCR/ABL* fusion gene on the Ph^1^ chromosome (not shown) that presents the usual 22q morphology. In spite of the complexity of these translocations, deletions adjacent to the t(9;22) breakpoint on the derivative chromosome 9 were not detected. Further characterization of the chimeric *BCR-ABL* transcript by QT-RT-PCR detected the b3a2 fusion transcript in all the patients. These results of cytogenetic and molecular analysis are summarized in Table 2. 

At present, all the patients are doing well in hematological and complete cytogenetic remission following standard dose imatinib treatment, except for patient (a) who died of congestive heart failure not related with imatinib treatment. Patients (d) and (e) showed complete disappearance of the fusion transcript after 71 and 86 months of follow-up, respectively. Patients (a)–(c) reduced the BCR-ABL transcripts levels in more than 2 logs (Table 2).

## 4. Discussion

In the present report we analyzed five patients with CML carrying complex Ph^1^ translocations involving various partner chromosomes by cytogenetics, FISH, and molecular methods. In each case, chromosomal translocations lead to a *BCR-ABL* fusion, as occurs in the standard t(9;22) translocation [2]. The third chromosome present in each of these variant translocations is known to be implicated in some cases of Ph-positive CML cases. Besides, the involvement of bands 3q21 (3 cases), 5q31 (2 cases), 7q32 (1 case), 8q24 (3 cases), and 10q22 (9 cases) had also been previously reported in other cases of CML [7]. Nevertheless, it becomes difficult to report the exact number of cases with such complex translocations due to the large amount of variability in cytogenetics nomenclature observed before ISCN, 2005.

Evaluation of the prognostic significance of these translocations has been analyzed in case reports or small series giving controversial results. However, it has been recently reported that patients with variant translocations have a similar prognosis to those with classical Ph^1^ translocations when treated with imatinib mesylate [8–10]. In our series, all the patients are at present in hematological and complete cytogenetic remission following standard-dose imatinib treatment after 12 to 86 months of follow-up. Regarding molecular remission, patients (d) and (e) showed complete disappearance of the fusion transcript after 71 and 86 months, respectively. Patients (a)–(c) did not reach complete disappearance of the fusion transcript but reduced the levels in more than 2 logs. Patient (a) showed a lighter reduction probably due to the complex karyotype at diagnosis.

Deletions of der(9) have been recognized in 10%–15% of patients in the chronic phase, being more frequently found in variant translocations. These deletions are thought to occur at the time of the Ph^1^ translocation and are known to be associated with a worse survival [11]. However, a recent study has suggested that imatinib mesylate may overcome the adverse prognostic significance of der(9) deletions [12]. In our study, none of the patients had a deletion of a sizable portion on the derivative chromosome 9. 

In conclusion, we described five low-frequency complex variant t(9;22) translocations representing 6% of the CML cases diagnosed in our center during approximately seven-year period. Despite low numbers, in our experience patients carrying complex Ph^1^ translocations do not differ significantly in hematological and clinical features from those with standard translocation.

## Figures and Tables

**Table 1:** Clinical characteristic of the patients. M: male, F: female, WBC: white blood cell count, PLT: platelet count, BM: bone marrow, Eo: eosinophils, Bso: basophils, HU: hydroxyurea, IFN*α*: interferon *α*, Auto: autologus, PBSC: peripheral blood stem cell transplantation.

Case	Sex/Age	WBC (10^9^/L)	PLT (10^9^/L)	BM Blasts (%)	BM Eo/Bso (%)	Phase at diagnosis	Treatment	Follow-up (months)
(a)	M/75	306	390	0	6/1	Chronic	HU, imatinib	30
(b)	F/50	671	408	2	11/2	Chronic	HU, imatinib	+12
(c)	F/59	123	250	0	—	Chronic	HU, imatinib	+18
(d)	M/56	431	222	0	5/0	Chronic	HU, auto-PBSC, IFN*α*, imatinib	+71
(e)	F/71	689	479	2	5/2	Chronic	HU, imatinib	+86

**Table 2:** Cytogenetic and molecular results.

Case	Karyotype	5′ ABL deletions	RT-PCR	BCR-ABL/ABL (%) diagnosis	BCR-ABL/ABL (%) after imatinib
(a)	46, XY, t(5;9;22)(q31;q34;q11)[5]/45, X, idem, −Y[4]/46, X, idem, +8[3]/46, XY, +8, t(5;9;22)[2]/46, XY, −4, t(5;9;22), −7, +der(X)? t(X;1)(p11.4;q21), +mar[6]	NO	b3a2	11.09	0.11
(b)	46, XX, t(9;22;10)(q34;q11;q22)[20]	NO	b3a2	1.75	0.014
(c)	46, XX, t(7;9;22)(q34;q11;q32)[25]	NO	b3a2	13.56	0.004
(d)	45, XY, t(3;9;22)(q21;q34;q11), −15[4]/46, XY, idem, +22[4]/46, XY[1]	NO	b3a2	11.06	Negative
(e)	46, XX, t(8;9;22)(q24.1;q34;q11)[20]	NO	b3a2	N.A	Negative
